# Enabling genomic surveillance from 30 years of linked English sentinel network data: The Wellcome Quinquagenarian (QQG) Biomedical Resource

**DOI:** 10.12688/wellcomeopenres.23653.1

**Published:** 2025-08-04

**Authors:** Simon de Lusignan, Praveen SebastianPillai, Omid Parvizi, Cecilia Okusi, Mark Joy, Shuma Banik, Fatima Batool, Katja Hoschler, Beatrix Kele, Angie Lackenby, Joanna Ellis, Richard Pebody, Conall Watson, Jamie Lopez Bernal, Maria Zambon

**Affiliations:** 1 Nuffield Department of Primary Health Care, University of Oxford, Oxford, Oxforshire, OX2 6GG, UK; 2 Royal College of General Practitioners, London, England, UK; 3 Respiratory Virus Unit, UK Health Security Agency, London, UK; 4 Immunisation and Vaccine Preventable Diseases Division, UK Health Security Agency, London, UK; 5 United Kingdom Health Security Agency, London, UK

**Keywords:** Influenza A, SARS-CoV-2, Respiratory Syncytial Viruses RSV respiratory Virus, H1N1, H3N2 Subtype Subtype, General Practitioners, Vaccine Efficacy, Influenza, Outcome Assessment, Health Care, Vaccines, Feasibility Studies, Genetic Code, Metadata, Primary Health Care, Genomics, United Kingdom

## Abstract

**Background:**

The World Health Organisation recommends integrating viral genome sequences and sentinel surveillance data. We report progress in linking clinical, virology, and sequence data to enable genomic surveillance of influenza, respiratory syncytial virus (RSV), and severe-acute-respiratory-syndrome coronavirus-2 (SARS-CoV-2).

**Methods:**

We linked individual-level clinical data from the Oxford-Royal College of General Practitioners (RCGP) Research and Surveillance Centre (RSC) sentinel network to virology results from the UK Health Security Agency (UKHSA) reference virology laboratory. We identify where publicly accessible repositories, the Global Initiative on Sharing All Influenza Data (GISAID), or others hold viral genome sequence data from test-positive cases. Our metadata also identifies test-negative controls contemporaneous to test-positive cases. We summarise the scope of data availability in the Wellcome Quinquagenarian (QQG) biomedical resource.

**Results:**

We report respiratory virus sampling for influenza, RSV, and SARS-CoV-2 between 1992 and 2023. Samples were collected from a nationally representative subset of RSC general practices participating in the virological surveillance programme.

QQG contains 13,665 positive influenza samples, 3,791 positive RSV samples, and 5,068 positive SARS-CoV2 samples. There were 2,819 sequenced influenza genomes, of which 97.1% were linked to clinical records, 1,251 sequenced RSV genomes of which 96.8 were linked to clinical records, and 2,486 sequenced SARS-CoV-2 genomes of which 98.9% were linked to clinical records.

**Conclusion:**

We have described the scale of QQG, created to enable genomic surveillance linked to clinical metadata to facilitate research on the impact of different viral variants on clinical outcomes, vaccine effectiveness, and therapeutic strategies.

## Introduction

The emergence of new viral variants can result in the further spread of disease and reduce the effectiveness of countermeasure programs, as was seen following the spread of drug-resistant influenza in 2008 ^1^ and during the COVID-19 pandemic. The recent introduction of vaccine and monoclonal antibody therapies for respiratory syncytial virus (RSV) will also require contemporary monitoring of viral diversity ^2^. To monitor such changes, the World Health Organisation’s (WHO) 10-year global genomic surveillance strategy recommends public health authorities integrate genetic sequence data (GSD) into disease surveillance ^3, 4^.

Integrating genomic and clinical data will enhance the genomic surveillance of viruses of public health significance ^5^. Timely virological surveillance can link viral gene sequence data with clinical characteristics of circulating strains, which, when further linked to vaccine exposure and disease burden data, enables estimates of vaccine effectiveness (VE) by viral variant, age, and severity of illness being reported. Genomic surveillance has already been implemented in acute and primary care settings for variants of SARS-CoV-2 with evidence of its utility during the pandemic period ^1, 6, 7^.

Establishing the Wellcome Quinquagenarian (QQG) resource for a range of seasonal respiratory viruses will provide closer to real-time evidence of the impact of countermeasures in a systematic and consistent framework, which can be scaled as needed with the emergence of significant variants.

We aim to create a biomedical resource that captures England's systematic genomic surveillance of influenza, RSV, and (SARS-CoV-2). Sentinel clinical surveillance started in the 1966–67 season ^8^. Current practice is built upon a longstanding collaboration between the Royal College of General Practitioners (RCGP) Research and Surveillance Centre (RSC) and the UK Health Security Agency (UKHSA) and its government public health agency predecessors, with the University of Oxford a more recent addition to this partnership ^9, 10^. Prospective combined virological and clinical surveillance for influenza began in the winter of 1992–3 and gradually increased its sophistication ^11, 12^. RSV detection was added in 2001, with sequencing of this virus added retrospectively ^13^. Detection of SARS-CoV-2 was added immediately in 2020 as part of an emergency response to the pandemic, with sequence analysis of positive samples added as routine from the start ^14^.

There has been no systematic curation or consolidation of these data; clinical, laboratory, and genomic data currently sit in separate repositories. The recently published Sudlow review, commissioned by England’s Chief Medical Officer articulates the barriers that arise from the UK’s complex and inefficient systems for managing and accessing health data and the potential role of major national public bodies with responsibility for or interest in health data to improve critical national infrastructure for data usage ^15^. In the 55 ^th^ (quinquagenarian) year of primary care sentinel surveillance we conceived creating a resource based on the combined clinical and virological data arising from a long-standing national community surveillance programme that could be interrogated by independent researchers ^16^. The resource was named the Wellcome Quinquagenarian (QQG) Biomedical Resource. Below, we describe its history, scope, and components.

## Methods

### Components of the Wellcome Quinquagenarian (QQG) biomedical resource

**Figure 1.**
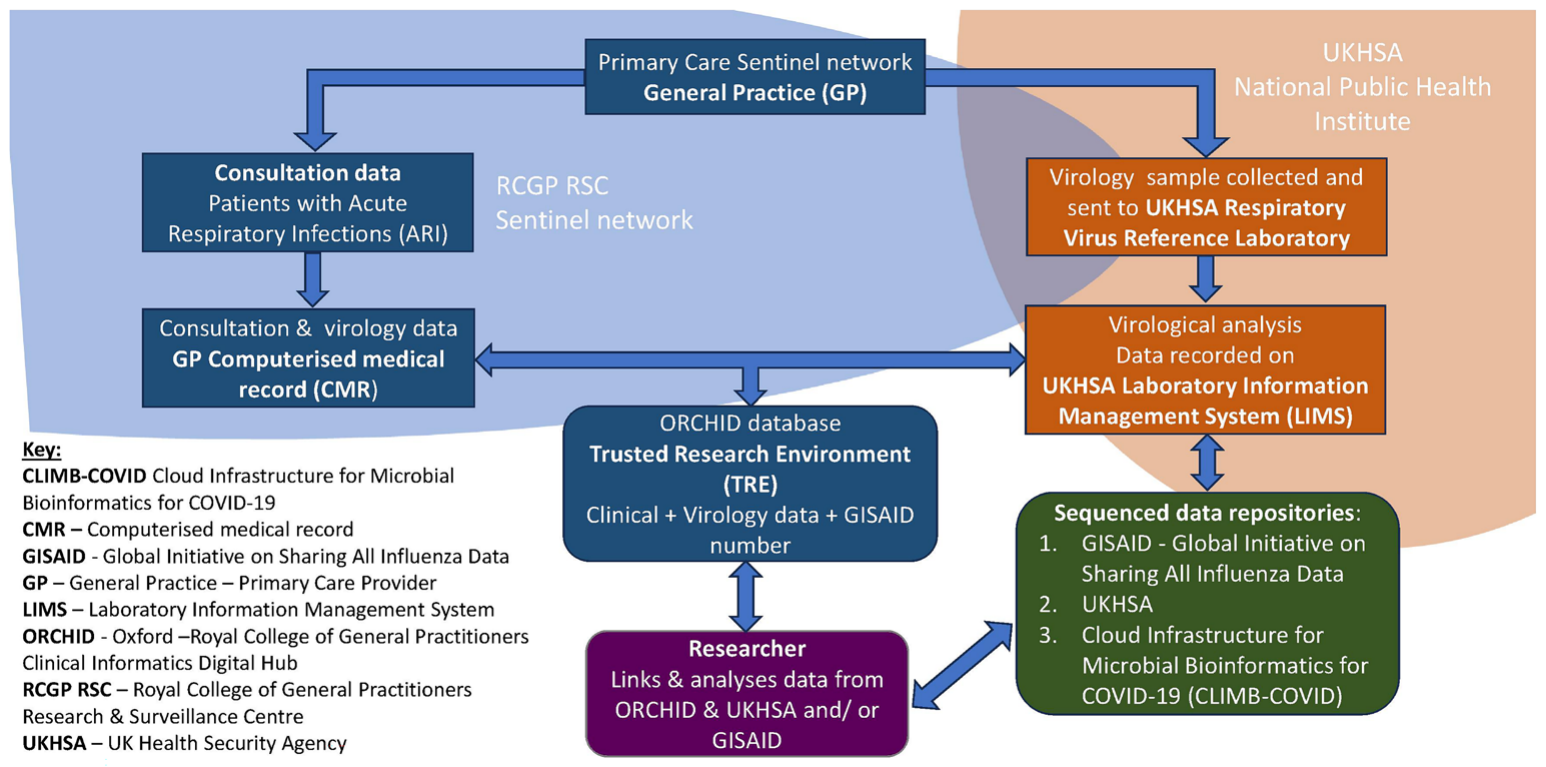
Schematic diagram of the federated data sources, where linked data sets do not physically leave their respective data stores, to create a resource for population-based genomic surveillance.

The three data components combined to make up the QQG biomedical resource were: (1) clinical data from the RSC, the English national primary care sentinel network, (2) virology data from UKHSA’s Respiratory Reference Virology Laboratory information system (LIMS) arising from sampling of a subset of cases for whom clinical data was recorded, and (3) virology sequence data stored in sequence data repositories, deposited by UKHSA or predecessors ( Figure 1).

### Data linkage, and virology sampling

The UK has a registration-based primary care system using a unique identifier (NHS number). This enabled the RSC to link an individual’s pseudonymised computerised medical record (CMR) data to other health systems and national data, making it readily usable for surveillance and research ^17^. At an individual level, pseudonymised patient records were routinely linked to a geographical location, and using the Lower Super Output Area (LSOA) from the Office of National Statistics (ONS) census which is used for reporting small geographical areas of between 1,000–3,000 people ^18^, hospital attendance and admission (Hospital Episode Statistics (HES) data) ^19^, and death data. The NHS Personal Demographics Service (PDS) ensures that the date of death is recorded in the GP record ^20^ and the Office of National Statistics (ONS) provides links to death certificate data ^21^.

Most pre-school and adult vaccinations were carried out in primary care, and where vaccine exposure happens elsewhere (mainly pharmacies and schools), records of these events were either directly transferred into the primary care record or the GP was notified. This transfer is routinely in place for influenza, RSV, and COVID-19 vaccines ^22^. NHS number was also used to link virology reference laboratory results to the GP CMR, each sample also had a laboratory information management system (LIMS) identifier (ID). The latter facilitated clinical, laboratory, and viral genomic sequence data linkage.

From the 1992–1993 winter season onwards, a subset of RSC practices collected virology samples from cases of influenza-like illness (ILI). Up to the COVID-19 pandemic year of 2020 this was a seasonal collection during the winter months. Virology sampling generally took place between the International Organisation for Standardisation (ISO) week 40 and week 20 of the following year. Reflecting the focus on influenza, clinically defined cases of influenza-like illness (ILI) were eligible for sampling ^23^. Whilst ILI was the RSC’s long-term clinical indicator for sampling new episodes of illness within 7 days of illness onset, clinical sampling was extended in 2012 to include acute bronchitis in children under 5 years old, coinciding with the first pilot of live attenuated influenza vaccine (LAIV) ^9^, though there had been longer-term interest in the association of acute bronchitis and winter pressures ^24^, and the importance of RSV in those who present with acute bronchitis ^25^. From 2020, as part of the pandemic response, sampling changed to become year-round and included any clinical presentation of an acute respiratory infection (ARI) ^10^, with larger numbers of samples collected (up to 1,000 per week) and a broader panel of viruses tested for 26.

Throughout the entire period, the RSC conducted virological sampling of the nasopharynx, using two swabs (one nasal and one throat) placed into a single vial of Virus Transport Medium (VTM), with samples sent through the postal system to the UKHSA reference laboratory. Most samples were taken by healthcare professionals, although intermittent patient self-swabbing was implemented before 2020 ^27^, and became a permanent parallel stream from 2020 ^28^.

### Respiratory virus laboratory analysis

Swabs collected in Virus Transport Media (VTM) were transported to the laboratory through the post at ambient temperature, with a mean time to arrival of 2-3 days ^12^. Each sample received was given a unique LIMS identifier and processed for the molecular detection of a range of viruses, with residual sample material stored at -80C.

Assays used for the detection and characterisation of influenza A and B inevitably changed over time, to take account of genetic drift in influenza and the evolution of molecular detection techniques (Figure S1). Techniques were based on the use of reverse transcription polymerase chain reactions (RT-PCR) for the detection of viral targets in different multiplex formats, updated regularly ^12, 29^.

The methodology for influenza genomic sequence reporting evolved from partial genome sequencing of the viral haemagglutinin (HA) gene using Sanger sequencing, then adding viral neuraminidase (NA) genes, and from 2009 onwards completing whole genome sequencing (WGS) of influenza using Illumina platforms. Molecular analysis of influenza was accompanied by phenotypic characterisation of selected virus isolates, including analysis of antiviral susceptibility to neuraminidase inhibitors based on the culture of virus isolates from residual VTM samples. This followed the recommendations of the WHO Global Influenza Surveillance and Response System (GISRS) for testing the antiviral susceptibility of influenza viruses ^30^. Scanning for altered antiviral susceptibility is now conducted using single nucleotide polymorphism (SNP) screening from WGS ^31^ to allow the identification of common resistance markers. RSV A and B PCR detection was targeted on the highly conserved regions of the genome, with little variation, with retrospective use of samples to generate whole genome sequences ^32, 33^.

SARS-CoV-2 detection also involved multiple target detection of conserved regions of the genome. These included the large open reading frame (ORF1ab) that encodes viral polyproteins, the E gene that encodes the envelope protein, and probes and primers to enable detection and amplification of these regions of the SARS-CoV-2 genome ^33^. Results were reported with RT-PCR cycle threshold (Ct) values provided for each assay target. RT-PCR virus assays with a cycle-threshold (Ct) value of under 40 were regarded as positive. In general, samples with a positive Ct value of <30 provided good-quality WGS data ^34, 35^.

### Repositories holding viral sequenced data

The Global Initiative on Sharing All Influenza Data (GISAID) has been the primary location used to hold influenza and RSV sequence data. GISAID was established in 2008 as a not-for-profit organisation to make sequenced data available for scientific study. Each set of sequenced data deposited has been provided a unique and permanent identifier ^36, 37^.

SARS-CoV-2 sequence data were deposited with the Cloud Infrastructure for Microbial Bioinformatics (CLIMB-COVID) developed by the COVID-19 Genomics UK Consortium (COG-ID) in response to the SARS-CoV-2 pandemic. The metadata captured included the date of sampling, geographical location, and sequence technology used ^38, 39^. Whilst this viral genome sequence repository was used, its overlap with GISAID deposition was beyond the scope of this paper; we include an inventory of RSC-derived data deposited in GISAID only. Sequence data before 2008 were stored locally within UKHSA and were excluded at this stage from our results.

### Linking process

The GISAID number was the primary key we used to link sequence data with reference virology laboratory data and clinical data for influenza and RSV. Viral genomic sequence data were deposited in GISAID (for influenza and RSV) and CLIMB-COVID (for SARS-CoV-2) by UKHSA. These data included the UKHSA LIMS number, which is a unique identifier (ID) for the virological sample. This enabled the linkage of GISAID data to reference virology laboratory data. Virological samples with the LIMS ID were also stored with the patient’s pseudonymised NHS number, the unique NHS identifier used throughout the health system, which facilitated additional linkage to the primary care CMR and other health system data.

We also set up a process to enable contemporaneous test-negative controls to be identified. The latter may be needed for any test-negative design (TND) vaccine effectiveness (VE) studies being undertaken ^40^. TND is commonly used to assess VE for a range of vaccines ^41, 42^.

### Data summary

A data summary will be placed online. The number of sequenced samples will also become part of the RSC’s Annual Report. How these data might be visualised is described (Figure S4 and S5).

## Results

### Sentinel network data

The RSC has grown in terms of size, scope, integration virological testing, and data linkage. When the RSC started sentinel surveillance in 1967, general practice members collected data on paper spreadsheets which were sent to the RCGP’s Birmingham Research Unit (BRU) for collation ^43^. From 1994 onwards, data flows were progressively computerised. The RSC leadership moved to the University of Surrey and in 2015, a new pseudonymised flow of data commenced ^44^, and subsequently to the University of Oxford with data flowing in 2021. Data were stored in the Oxford Royal College of General Practitioners Clinical Informatics Digital Hub (ORCHID) database, hosted by the Nuffield Department of Primary Care Health Sciences, University of Oxford, a trusted research environment (TRE) ^10^.

Pseudonymisation used an NHS England-approved method allowing linkage to other health datasets ^45^. We used a non-reversible approach, the Secure Hash Algorithm 512 (SHA 512). SHA 512 is a commonly used approach. We convert the NHS number into a fixed-size string. Each output produces a SHA-512 length of 512 bits (64 bytes). We added a salt before hashing to make data more secure. Between 1967 and 1997, aggregated clinical data were collected from paper records onto spreadsheets in individual general practices, forwarded to BRU, and stored on a Microsoft Access database (BRU-Access). In 1994, the first Computerised Medical Record (CMR) data started flowing to BRU and was stored on a Microsoft SQL database (BRU-SQL). In 2015, BRU-SQL was replaced by the University of Surrey-based SQL Real World Evidence (RWE) database. Retrospective data from 2004 were included in this new database, to coincide with the date when pay-for-performance for chronic disease management started in primary care ^46^ and GP CMR systems were linked to pathology laboratories. The net effect of the Quality and Outcomes Framework (QOF), was to incentivize improved data recording in primary care, leading to better data quality particularly associated with cardiovascular comorbidities. The role of electronic laboratory links in enhancing data quality also started to be recognised at this time ^47^. Such links enable the seamless transfer of pathology data into primary care records, ensuring completeness and accuracy.

**Figure 2.**
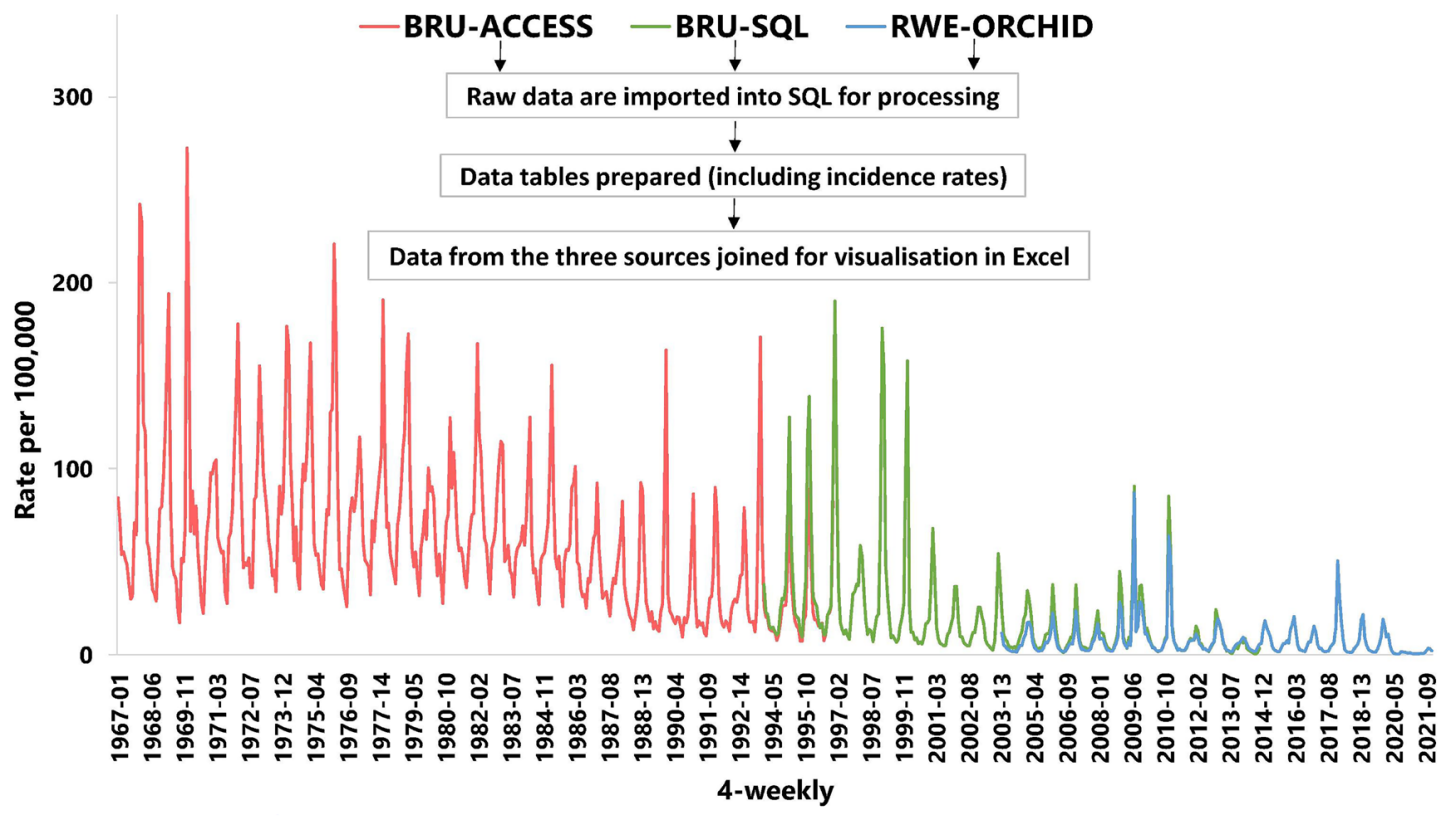
Mean 4-weekly incidence of influenza-like illness (ILI) across different RSC clinical data sources. Data were originally collected on a spreadsheet and collated in an access database (BRU-Access, red), then collected as a computerised medical record (CMR) extract (BRU-SQL, green). More recently individual pseudonymised CMR data were collected into the real-world evidence (RWE) and then ORCHID servers (blue). There is overlap of data sources between 1994–1997, and from 2004 onwards within the RWE & ORCHID and BRU-SQL systems.

Together, QOF and electronic laboratory links have contributed to the high quality of data in UK general practice, making it suitable for research and quality improvement initiatives ^48^. This database moved to Oxford in 2021 and was renamed ORCHID. Figure 2 illustrates how longitudinal ILI data can be combined, the re-extraction of BRU data is very similar to that previously reported ^9^.

**Figure 3.**
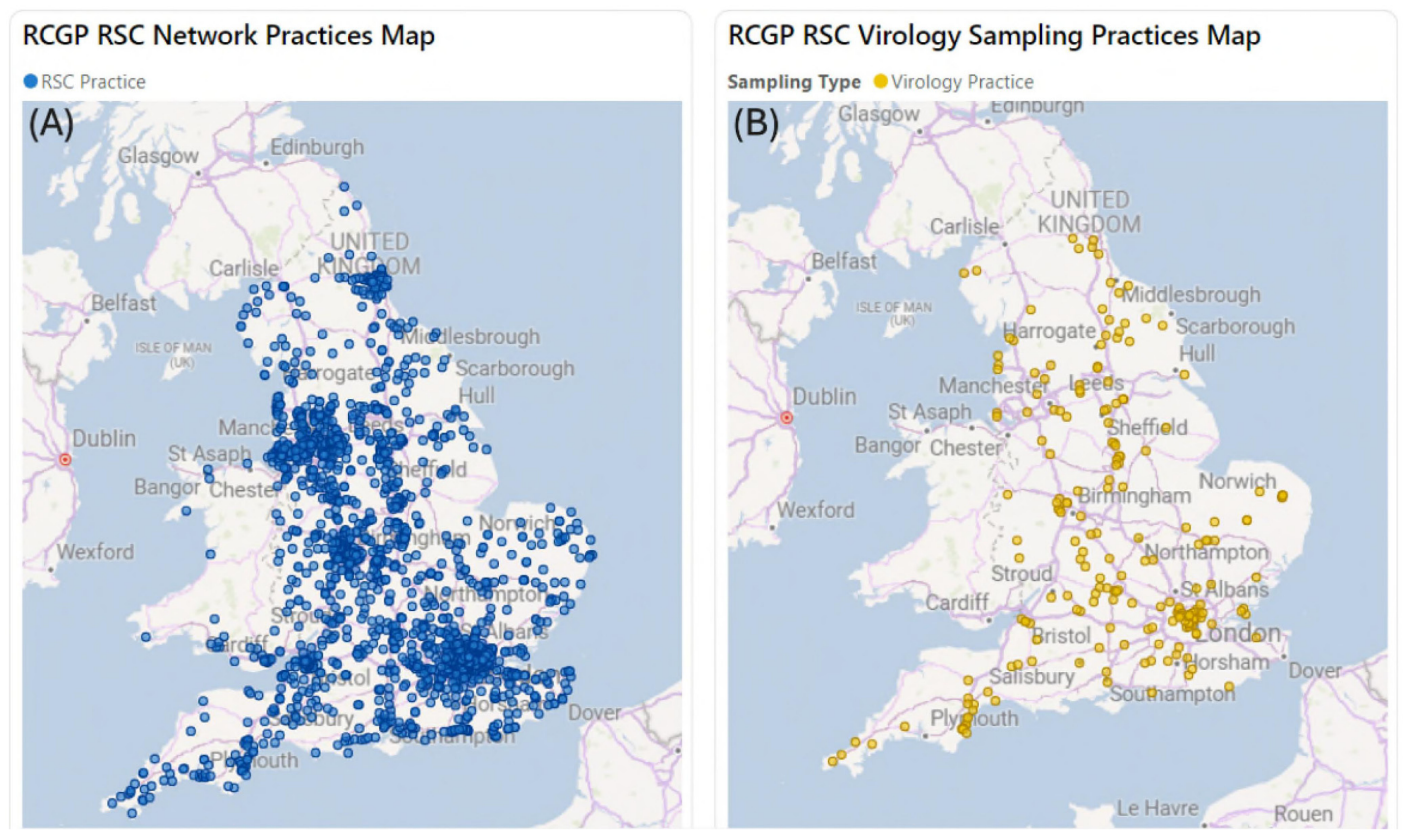
( **A**) General practices in the UK of the English primary care sentinel network on the 19 ^th^ of November 2024. ( **B**) Virology sampling practices providing at least one sample during the 2023 calendar year.

The RSC in 1977 had 39 general practices, representing a patient population of around 200,000 ^28^; rising to over 100 practices covering a population of over 1 million in 2016 ^29^; then growing to 1,879 practices, a population of 17 million, 31% of the English national population in 2021 ^49^. The RSC has recruited its practices and the subset of virology sampling practices to be nationally representative ( Figure 3).

### Respiratory virology reference laboratory data

**Figure 4.**
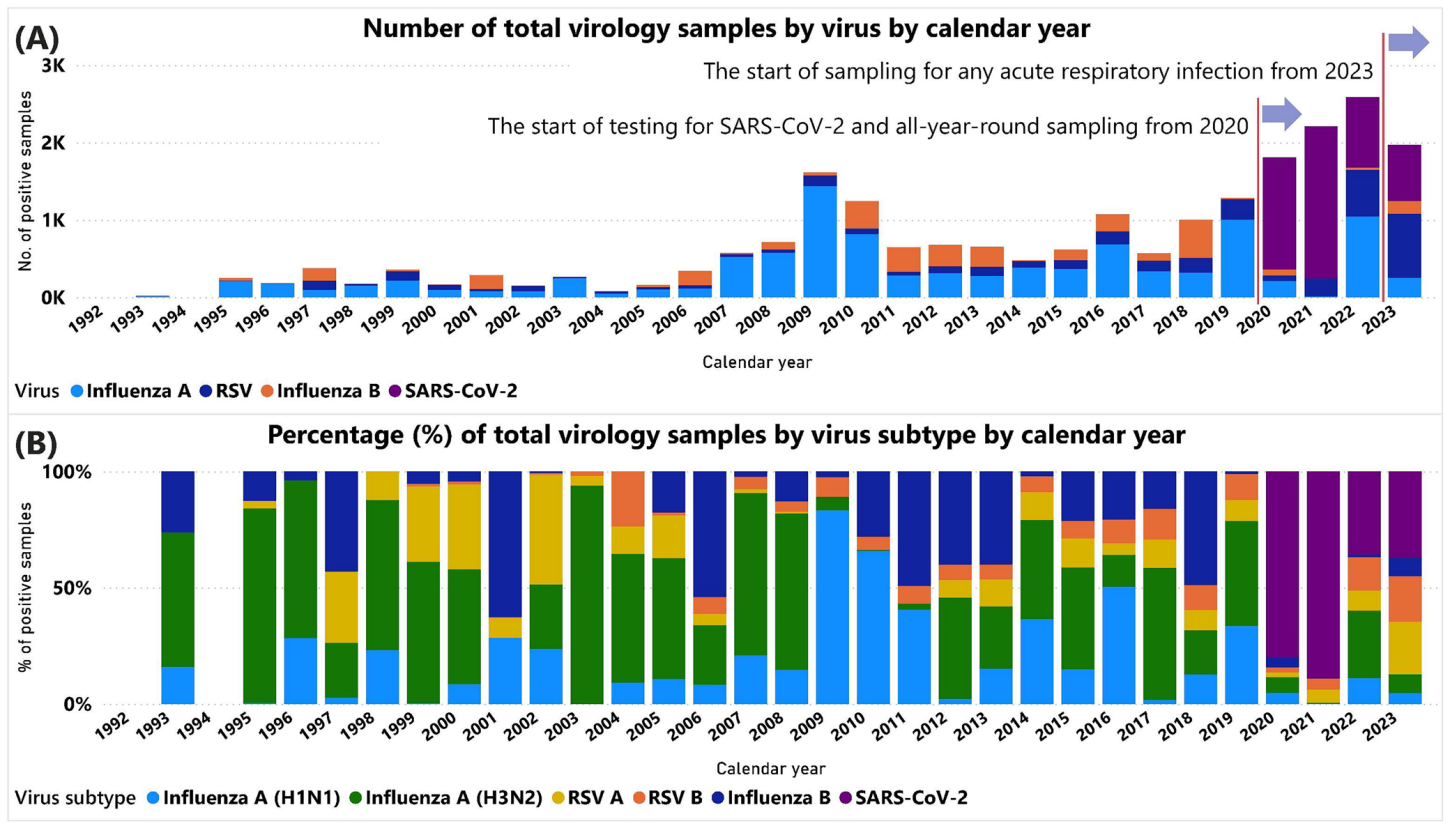
Details of positive respiratory virus data analysed by UKHSA or predecessor agencies between 1992 and 2023. ( **A**) The number of positive samples by virus subtype by year. ( **B**) The percentage of the total number of positive samples tested by virus subtype by year.

We report on the availability of influenza, RSV, and SARS-CoV-2 sequence data from virology swab samples where these viruses were detected. Before the 2009 influenza pandemic caused by H1N1, the number of positive samples for influenza and RSV combined was generally between 100 and 700 over the course of the winter seasons, the proportion of positive varying by week across the epidemic period. During the peak of the ILI consultation rate periods, normally lasting 6–8 weeks, the rate of influenza positivity increased up to 50–60% from <5% before the onset of sustained influenza circulation. In 2009 the number of positive influenza samples rose to over 1,600 and steadily increased thereafter. From 2020, testing for SARS-CoV-2 commenced with a switch to all-year-round sampling from 2021 onwards ( Figure 4).

Between 2002 and 2023, influenza A viruses, either H1N1 or H3N2 were the predominant influenza viruses detected and typically co-circulated in most seasons with varying proportions. The H1N1 (H1) subtype caused a pandemic in 2009 (H1N1pdm09), replacing the previous seasonal H1N1 virus completely during 2009, with the highest proportion of H1N1(pdm09) detected in the winter periods (2010–2012) immediately following the pandemic of 2009 ( Figure 4B). There has been co-circulation of both Victoria and Yamagata lineages of influenza B over these 20 years, but the latter influenza B subtype has not been detected since 2019 through RSC sampling. The detection of transmissible influenza resistance to oseltamivir in circulating seasonal H1N1 before the 2009 H1N1 pandemic was an example of the clinical utility of testing the circulating influenza A subtypes for antiviral susceptibility, providing an estimate of the proportion of circulating viruses with altered antiviral susceptibility ^50, 51^. A sporadic case of influenza A H1N2v zoonotic infection from swine was also detected in 2023 through the RSC virological swabbing programme in an area of England with the densest swine population. Part of the incident response to this unexpected detection event included an escalation of virological sampling of cases of ARI in the surrounding localities ^52^.

Since 2003, similar rates of RSV A and B subtypes have been identified in each season, with a gradual expansion of sampling. Between 1997 when RSV testing was introduced, and 2003 RSV A predominated. Most recently, in 2023 the RSC collected 827 positive RSV samples: 53% (n=442) RSV A and 47% (n=385) RSV B. RSV containing samples from 2008 onwards have been used for WGS analysis, if technically suitable, to underpin studies of RSV viral diversity in England.

SARS-CoV-2 testing of virological samples was included from March 2020 onwards, as part of the pandemic response, but only samples collected from symptomatic patients were included, with ILI as the main clinical indicator at the time. A total of 5,068 positive samples were collected, 67% (n=2,406) in the first two years of the pandemic in 2020 and 2021. These samples were all submitted for WGS analysis, if technically suitable.

### GISAID-held viral genomic sequence data

Since 1992, 22,529 submitted samples have been positive for either influenza A or B, RSV or SARS-CoV-2 of which the majority 60.7% (n=13,665) were influenza, reflecting the origin and purpose of the virological testing programme, intended as a method of monitoring the circulation of influenza A and B in the community. A smaller proportion were RSV {16.8% (n=3,791)} and 22.5% (n=5,068) were SARS-CoV-2 (Table S2). Just under a third of these samples overall 29% (n=6,556) underwent whole genome sequencing to monitor viral diversity and provide information for the selection of samples for virus isolation and antigenic analysis, selected mainly on technical suitability for sequence analysis (sufficient sample, well preserved with adequate viral load).

Over 100 H1N1 WGS were obtained in 2009 as part of a scaled-up response to the 2009 pandemic at the time, this represented a major increase in viral sequencing activity, with the use of sequence data to track viral evolution during the early course of the 2009 pandemic. Sequence analysis was reduced between 2010 to 2013, following the de-escalation of the pandemic response. From 2014 onwards, there were increasing numbers of influenza whole genome sequences (WGS) generated each year, reflecting the gradual improvement in higher throughput laboratory sequencing methodologies, up to several hundred of each influenza subtype in the years before the pandemic of 2020. The number of influenza whole genome sequences generated reduced to 69 in 2020 and 6 in 2021, as a result of interrupted influenza transmission arising from pandemic lockdown measures ( Table 1). Between 2009 to 2023, a total of 2,819 influenza WGS sequences were generated ( Table 1). 97.1% of these can be linked to RSC clinical data.

**Table 1.** Influenza sequence data held on GISAID with linkage to the clinical record.

	UKHSA Influenza sequences stored in GISAID *n*	RSC Influenza sequences stored in GISAID by subtype				Linkage of RSC sequences to the individual patient record *n (%)*
Year		Influenza A (HIN1) *n*	Influenza A (H3N2) *n*	Influenza B *n*	Total Influenza *n (%)*	
2009	507	109	0	0	109 (21.5)	105 (96.3)
2010	53	12	0	1	13 (24.5)	12 (92.3)
2011	14	1	2	0	3 (21.4)	2 (66.6)
2012	13	1	1	0	2 (15.4)	2 (100)
2013	10	3	0	1	4 (40)	4 (100)
2014	193	13	102	2	117 (60.6)	99 (84.6)
2015	224	22	67	14	103 (46)	96 (93.2)
2016	619	177	93	106	376 (60.7)	346 (92.0)
2017	785	10	173	91	274 (34.9)	266 (97.0)
2018	1,021	52	69	285	406 (39.8)	402 (99.0)
2019	1,285	100	314	10	424 (33)	423 (99.7)
2020	205	20	30	19	69 (33.7)	68 (98.5)
2021	194	1	3	2	6 (3.1)	6 (100)
2022	2,970	22	538	162	722 (24.3)	719 (99.5)
2023	576	40	57	94	191 (33.2)	190 (99.4)
Total	8,670	583	1,449	787	2,819 (32.5)	2,740 (97.1)
% by virus subtype		20.7	51.4	27.9		

Over half of all influenza sequences derived from RSC sampling stored on GISAID since 2009 were the influenza A (H3N2) subtype (51.4%, n=1,449), reflecting the dominance of circulation of this subtype in England over the time period ( Figure 4B). The H1N1 subtype of influenza A contributed (20.7%, n=583), with influenza B (27.9%, n=787). 33% of all UKHSA influenza sequence data stored on GISAID are represented by samples collected by the RSC, comprising a geographically representative sample of viruses circulating in the community over this period of time.

RSV viral genome sequencing (WGS) was undertaken retrospectively using RSC samples archived since 2008. ( Table 2), using a variety of sequencing methodologies, (described in Talts et al 2023). These were mainly RSV B (59.2%, n=741), with the remainder RSV A (41%, n=510). The number of RSV-positive samples increased in recent years, reflecting the increased sampling of younger age groups, following the introduction of the LAIV influenza vaccine in 2013/14, with the exception during the COVID-19 pandemic years. A large proportion of RSV samples with WGS deposited in GISAID (96.8%) could be linked to clinical records. 75% of all UKHSA RSV sequence data stored on GISAID are represented by data from samples collected by the RSC, and a detailed phylogenetic analysis of RSV strain diversity over this period is underway.

**Table 2.** RSV sequence data held on GISAID with linkage to the clinical record.

	UKHSA RSV sequences stored in GISAID *n*	RSC RSV sequences stored in GISAID by subtype			Linkage of RSC sequences to the individual patient record *n (%)*
Year		RSVA *n*	RSVB *n*	Total RSV *n (%)*	
2008	8	0	8	8 (100)	8 (100)
2009	23	2	21	23 (100)	19 (82.6)
2010	16	1	15	16 (100)	13 (81.3)
2011	7	0	7	7 (100)	1 (14.3)
2012	32	21	11	32 (100)	27 (84.3)
2013	22	13	7	20 (90.9)	18 (90.0)
2014	21	14	7	21 (100)	19 (90.4)
2015	15	6	8	14 (93.3)	10 (71.4)
2016	24	11	10	21 (87.5)	18 (85.7)
2017	16	13	3	16 (100)	16 (100)
2018	25	8	17	25 (100)	25 (100)
2019	243	75	91	166 (68.3)	166 (100)
2020	51	6	20	26 (51)	26 (100)
2021	379	95	68	163 (43)	161 (98.7)
2022	581	187	325	512 (88.1)	510 (99.6)
2023	30	58	123	181 (603.3)	175 (96.7)
Total	1,658	510	741	1,251 (75.5)	1,212 (96.8)
% by virus subtype		40.8	59.2		

Only 3.1% of all UKHSA SARS-CoV-2 sequence data stored on GISAID are represented by data from samples collected by the RSC (N=2,486) ( Table 3), reflecting the massive scale-up of community sampling and viral WGS sequencing in the UK over the pandemic period. The period of maximum sequencing occurred in 2021, (N=1,365) ( Table 3), with gradual de-escalation since this time period. 98.9% of these sequenced samples could be linked to their clinical record providing the most complete linkage of the three viruses included in our analysis. During this period of time, the waves of different SARS-CoV-2 viral variants could be seen (data not seen).

**Table 3.** SARS CoV-2 sequence data held on GISAID with linkage to the clinical record.

Year	UKHSA SARS-CoV-2 sequences stored in GISAID *n*	RSC SARS-CoV-2 sequence stored in GISAID *n (%)*	Linkage of RSC sequences to the individual patient record *n (%)*
2020	9,332	630 (6.8)	619 (97.9)
2021	54,201	1,365 (2.5)	1,354 (99.8)
2022	15,123	454 (3)	451 (99.3)
2023	1,034	37 (3.6)	37 (100)
Total	79,690	2,486	2,461 (98.9)

Table 4 provides a summary overview of all UKHSA influenza, RSV and SARS-CoV-2 samples sequenced from 2008 to 2023 held in GISAID, over 97% of all RSC samples received at the UKHSA laboratory were linked to a clinical record.

**Table 4.** Summary of the number of RSC sequences in GISAID with the percentage of total virology samples sequenced.

Year	Influenza A (HIN1) *n (%)*	Influenza A (H3N2) *n (%)*	Influenza B *n (%)*	Total Influenza *n (%)*	RSVA *n (%)*	RSVB *n (%)*	Total RSV *n (%)*	SARS-CoV-2 *n (%)*
2008 *	-	-	-	-	0 (0)	8 (25.8)	8 (21.6)	0 (0)
2009	109 (8.1)	0 (0)	0 (0)	109 (7.4)	2 (100)	21 (15.8)	23 (17)	0 (0)
2010	12 (1.5)	0 (0)	1 (0.3)	13 (1.1)	1 (50)	15 (21.7)	16 (22.5)	0 (0)
2011	1 (0.4)	2 (11.8)	0 (0)	3 (0.5)	0 (0)	7 (14)	7 (14)	0 (0)
2012	1 (6.7)	1 (0.3)	0 (0)	2 (0.3)	21 (41.2)	11 (24.4)	32 (33.3)	0 (0)
2013	3 (3)	0 (0)	1 (0.4)	4 (0.7)	13 (17.1)	7 (16.7)	20 (16.9)	0 (0)
2014	13 (7.4)	102 (49.5)	2 (20)	117 (29.8)	14 (24.1)	7 (21.2)	21 (23.1)	0 (0)
2015	22 (23.9)	67 (24.6)	14 (10.5)	103 (20.7)	6 (7.8)	8 (17.4)	14 (11.4)	0 (0)
2016	177 (32.7)	93 (62.8)	106 (47.3)	376 (41.1)	11 (21.2)	10 (9.1)	21 (13)	0 (0)
2017	10 (100)	173 (53.4)	91 (98.9)	274 (64.3)	13 (18.8)	3 (4)	16 (11.1)	0 (0)
2018	52 (40.6)	69 (36.5)	285 (58.2)	406 (50.3)	8 (9)	17 (15.7)	25 (12.7)	0 (0)
2019	100 (23.4)	314 (54.4)	10 (71.4)	424 (41.6)	75 (65.2)	91 (62.8)	166 (63.8)	0 (0)
2020	20 (23)	30 (24.6)	19 (24.7)	69 (24.1)	6 (16.7)	20 (52.6)	26 (35.1)	630 (43.7)
2021	1 (25)	3 (33.3)	2 (66.7)	6 (37.5)	95 (76)	68 (65.4)	163 (71.2)	1,365 (69.6)
2022	22 (7.5)	538 (71.4)	162 (558.6)	722 (67.2)	187 (82.7)	325 (87.4)	512 (85.6)	454 (48.7)
2023	40 (43.5)	57 (35.4)	94 (59.1)	191 (46.4)	58 (13.1)	123 (31.9)	181 (21.9)	37 (5)
Total	583 (11.9)	1,449 (26)	787 (24.6)	2,819 (20.6)	510 (26.7)	741 (39.5)	1,251 (33)	2,486 (49)

* Influenza virology sample and sequence data are not available in 2008.

## Discussion

### Principal findings

There is international acceptance of the importance of genomic surveillance ^53, 54^. With calls for a global network of laboratories generating sequence data ^55^. We have demonstrated how clinical records, virology results, and viral genome sequences obtained from sentinel surveillance programmes can be linked up in a systematic and consistent manner retrospectively, with pseudonymised data being made available for independent analysis. Going forward we are building community-based surveillance systems which are scalable for responses needed during pandemic periods, with intrinsic sequence data linkage to clinical metadata, to provide the analytical capability to rapidly assess circulating virus diversity against the outcome of interventions.

We have reported the number of samples collected since 1993, but focussed on samples with sequenced whole viral genomes collected from the primary care sentinel network and stored within GISAID. There are RSV and influenza positive samples from 2008/9 and SARS-CoV-2 since 2020. There is a high level (over 97%) of contemporary linkage to clinical records. This paper has reported the minimum sample numbers with complete data sets currently available for research.

Virological samples with or without virus detection, linked to clinical metadata, including patient age, date of sampling, vaccine exposure, can be analysed against clinical outcomes, including information about viral sequence coming from samples which have successfully undergone WGS, with data stored in GISAID from 2008. This inventory provides a rich and unique data repository arising from a longstanding, national community surveillance programme.

There have been some projects that have created complex performance federated environments for genomic surveillance ^56^, however, considerable care is required to ensure that these are privacy preserving environments, as we have developed in this project, and will continue prospectively for these three respiratory viruses ^57, 58^. The UK has an overall initiative in ARI genomic surveillance ^59^. A Scottish study using 150 influenza A (H3N2) linked clinical and genomic sequence data was able to draw epidemiological insights ^60^. Little appears to have been completed for RSV yet, though work is in progress and wastewater analyses have been conducted ^61^. The introduction of public health interventions such as RSV vaccine programmes emphasise the need for systematic genomic surveillance of this virus.

### Implications of the findings

The RSC, a longstanding community surveillance programme is developing the capability for sustained genomic surveillance of viruses of public health significance to build a scalable system for pandemic and interpandemic monitoring, linking clinical disease information to virological detection and assessment of viral diversity. Such a system is needed to provide analytical capability to monitor the effectiveness of the increasingly complex vaccine delivery programmes for influenza, RSV and SARS-CoV-2, across different segments of the population.

### Comparison with the literature

The widespread testing and need for rapid data access, meant large numbers of WGS were available for COVID-19. Utilisation of information was of clear clinical benefit ^62, 63^. The smaller number of SARS-CoV-2 WGS in the RSC are important for assessing the timeliness of detection through sentinel surveillance, compared with large scale clinical testing, providing valuable information about potential delays arising in detection of emerging viruses, and intensity of sampling needed for rapid detection of new variants. Long-term follow-up of the sequelae of SARS-CoV-2 infection, for example, people with long covid who can readily be identified from GP records ^64^ is also a valuable asset for assessment of clinical outcomes. Our findings around the disappearance of the influenza B Yamagata lineage has also been reported internationally ^65^.

### Strengths and limitations

The strength of these resources is the strength and longevity of the over 57-year RCGP-UKHSA partnership, relatively newly reinforced by Oxford in 2018. Over this period there was an emphasis on high-quality computerised medical record (CMR) quality, with virology sampling since the 1992/1993 winter, with viral WGS sequence data deposited in GISAID since 2008. This partnership has enabled us to create this unique resource. Some of its collections, such as an uninterrupted series of RSV viruses since 2008 prior to the introduction of a vaccination programme in 2024 will provide important insights into viral evolution when uncontested by vaccination and can be used as a comparator to viral evolution under vaccine pressure from 2024 onwards.

The main limitations of our work were the scope of our clinical data, sampling and sequencing, and the lack of federation for searching and analysing these data systematically, including for the provision of permissions to use this data asset. Our clinical data were routinely collected into primary care CMR and coded into those records, and inevitably there will have been data quality issues. The criteria for sampling changed over time, samples from 2023 can be from those with any ARI, and samples were collected all year round from 2020. Potential users will need to apply separately to each organisation for permission to use its data, even though we will create a single webpage through which to do this.

### Further research

Research using these data will strengthen the case for their further development. The growing demand for enhanced analytical capabilities may drive the sequencing of additional virology samples and would increase the statistical power of analysis. Integration with other repositories could also be explored.

How best to federate these data and permission to use them is the critical next step to promote their usability. Given the volume of sequence data, such a federated approach will need to include a high-performance computing environment. Given the policy constraints of processing health data outside NHS England-approved Secure Data Environments ^66^, UKHSA’s approved secure data environment, the Enterprise Data Analytics Platform (EDAP), and other options are being explored ^67, 68^.

## Conclusion

This paper describes the progress made to enable English primary care sentinel data and National Public Health Institute viral sequence genomic data to be available to enable independent analysis. The limitation of this work is the limited range of years of genomic surveillance data available over the 57-year history of the RSC, the lack of a single repository of these data, and the federated environment. However, it remains an achievement that of over 22,000 virology samples nearly 7,000 of these have sequenced data available for use in GISAID, with high linkage rates of sequenced genomes to RSC clinical data. We have undertaken ambitious strides towards enabling genomic surveillance.

## Ethics approval

The creation of the biomedical resource was approved by Health Research Authority (HRA) North East - Newcastle & North Tyneside 2 Research Ethics Committee (REC) Research Application System (IRAS) No 3288330, REC reference is 23/NE/0155, 24th August 2023.

## Data Availability

The Oxford Royal College of General Practitioners Research and Surveillance Centre (Oxford-RCGP RSC) provides access to routinely collected primary care electronic health records from a sentinel network of over 2,000 practices and 19 million patients. The dataset has expanded in size and scope since 1967. Since 1992, biosamples have undergone viral diagnostic testing at the UK Health Security Agency (UKHSA) reference laboratories, with viral genomic sequencing available since 2008. These data can be linked to other datasets, such as hospital records. While viral genomic sequences deposited in platforms such as GISAID are publicly available, they are not linked to clinical data. Linkage between clinical records and viral genomic data requires pseudonymisation to protect patient confidentiality. Analysis involving potentially identifiable, pseudonymised data must be conducted within the secure trusted research environment, ORCHID. De-identified datasets may be extracted to an open environment following statistical disclosure control and with appropriate ethical approvals. Access to RSC data is restricted due to ethical and data governance requirements. The dataset includes sensitive health information, and public sharing is not permitted. The University of Oxford’s Research Ethics Committee, along with the RCGP and UKHSA (as joint data controllers), have approved these data governance protocols. Researchers may apply to access the RSC dataset through a formal process, which includes: -Submission of a study protocol for review, -Induction onto University of Oxford IT systems (following initial approvals), -Specification of data requirements and demonstration of compliance with information governance standards for any data transfers to open environments. Research studies typically require approval from the University of Oxford, RCGP, UKHSA, and, where applicable, the Health Research Authority (HRA). The review process generally takes 21 working days, and pre-grant submission approvals can be obtained. For details on how to apply and current timelines, please visit the website - https://www.phc.ox.ac.uk/intranet/better-workplace-groups-committees-open-meetings/primdisc-committee-1.n
