# Supplementary material for: Enabling genomic surveillance from 30 years of linked English sentinel network data: The Wellcome Quinquagenarian (QQG) Biomedical Resource
Source: Wellcome Open Res. 2025 Aug 4;10:411. [Version 1] doi: 10.12688/wellcomeopenres.23653.1 (PMC12749565; doi:10.12688/wellcomeopenres.23653.1)
Supplement: Peer review history

# Reviewer report: Reviewer response for version 1

**DOI:** 10.21956/wellcomeopenres.26093.r139932

**Published:** 2025-12-29

**Authors:** Steven C. Holland

**Affiliations:** 1 Arizona State University, Tempe, AZ, USA

The article by de Lusignan et al. describes the linking of clinical, virological, and sequencing data obtained from multiple, separate repositories. At the original repository, some of this data contains PII and other sensitive information, requiring specialized and limited access. By providing a single access point and deidentifying patient information, this project broadens data availability to additional researchers. This allows further use of this data in future epidemiological and genomic studies. In this report, authors linked samples from 1992 through 2023 for influenza, RSV, and SARS-CoV-2 refining our virological surveillance over 30 years.

 In the manuscript, the authors describe curation methodology, provide illustrative example queries of database usage (e.g. Weekly incidence rate, geographic data mapping, subtype abundances), and discuss this database comparative to similar projects. Further, database access instructions are provided, to allow for broader access.

 All analyses and descriptions have been performed at a breadth and depth sufficient for further Indexing. 

 Minor recommendations for further clarity would include:

 - Describing the full extent of what information is included in each database. Researchers unfamiliar with each database may not be familiar with the data contained therein. If the list of tracked values is expansive for a particular database, then this may be included in a supplemental, rather than the text itself.

 - HIN1 is used in a couple tables, I believe this is just a transcriptive error of H1N1, but should be corrected, or defined if correct.

Is the work clearly and accurately presented and does it cite the current literature?

Yes

If applicable, is the statistical analysis and its interpretation appropriate?

Yes

Are all the source data underlying the results available to ensure full reproducibility?

Yes

Is the study design appropriate and is the work technically sound?

Yes

Are the conclusions drawn adequately supported by the results?

Yes

Are sufficient details of methods and analysis provided to allow replication by others?

Yes

Reviewer Expertise:

Molecular biology, virology, genomic epidemiology

I confirm that I have read this submission and believe that I have an appropriate level of expertise to confirm that it is of an acceptable scientific standard.

# Reviewer report: Reviewer response for version 1

**DOI:** 10.21956/wellcomeopenres.26093.r129833

**Published:** 2025-09-04

**Authors:** Rakesh K Mishra

**Affiliations:** 1 Tata Institute for Genetics and Society, Bengaluru, Karnataka, India

This is very useful study that links metadata with the evolving variants. Such comprehensive and long-term study provide information that can have predictive value for clinical aspects of new variants like disease severity, infectivity, immune escape, etc. It also adds value to effective genomic surveillance of pathogens. Furthermore, with the help of such information, environmental surveillance can also have greater utility. considering these aspects, I recommend indexing of this manuscript.

Is the work clearly and accurately presented and does it cite the current literature?

Yes

If applicable, is the statistical analysis and its interpretation appropriate?

Yes

Are all the source data underlying the results available to ensure full reproducibility?

Yes

Is the study design appropriate and is the work technically sound?

Yes

Are the conclusions drawn adequately supported by the results?

Yes

Are sufficient details of methods and analysis provided to allow replication by others?

Yes

Reviewer Expertise:

NA

I confirm that I have read this submission and believe that I have an appropriate level of expertise to confirm that it is of an acceptable scientific standard.
